# Editorial: Clinical scope of micronutrients in human viral infections

**DOI:** 10.3389/fnut.2023.1258886

**Published:** 2023-08-11

**Authors:** Benjamin Rakotoambinina, Laurent Hiffler

**Affiliations:** ^1^Lab LRI (Laboratory Radio Isotopes) Division of Isotopic Medicine, Pediatric and Adult Physiology, University of Antananarivo, Antananarivo, Madagascar; ^2^Cellular Nutrition Research, Lagny sur Marne, France

**Keywords:** viruses, micronutrients, immune response, inflammatory signaling, redox homeostasis, vitamin D, COVID-19, HIV

## Introduction

A broad spectrum of micronutrients (vitamins and trace elements) are involved in host processes during viral infections (VI). Upon entering the target cell, viruses develop strategies to hijack the host metabolism in the cytosol (1) and in organelles (2, 3) for the replication of their own genome, thus weakening host responses, which is a factor in poorer outcomes. A depletion of the host micronutrient stores and redox imbalance may ensue, prompting supplementation.

Several micronutrients can exert an immunomodulatory effect on the innate component by enhancing the type I interferon response, which acts as the guardians at the front-line for planning virus-killing defense (4, 5). Some micronutrients then act in the adaptive pathways by generating antibodies (6). Micronutrients are differentially credited with anti-inflammatory (7, 8) or antioxidant (9–11) and antiviral effects (12–14).

These promising experiments have prompted clinical studies using micronutrient supplements. Far from being always consistent, clinical evidence still remains elusive (15, 16).

We gathered clinical studies focusing on micronutrients in VI (DNA/RNA) and extended it to bench-to-bedside research (virology, immunology, and bioinformatics) of clinical interest.

## Results

An interactive peer review process involving 129 experts worldwide resulted in four articles.

Wang et al. conducted a systematic review/meta-analysis of the mean differences (MD) in serum vitamin D (sVitD-level) and prevalence of vitamin D deficiency (VDD) between HIV-infected subjects and non-HIV-controls. Subjects' characteristics and Highly Anti-Retroviral-Therapy (HART) were analyzed in 15 out of 3,184 initial studies.

The global analysis revealed significant MD in *VDD prevalence* with an overall odds ratio [OR, 95% CI, 1.502 (1.023–2.205)] for HIV vs. the control group.

The analysis of characteristics subgroups showed a significant ORs (95% CI) regardless of item (i.e., age over 40, latitude <40, BMI <25, and subgroup only on HART).

The *overall MD* (sVitD-level) between HIV and controls was −2.567 (95% CI, −5.976 to 0.843; *p* = 0.140), visualized on a worldwide map of included studies. The subgroup analyses showed a significant OR (95% CI) in overall MD between HIV and control groups for age category, latitude <40, and HART-naïve HIV.

This quantitative approach highlights that HIV infection is prone to VDD according to a risk-scale (BMI, latitude, and HART therapy interacting with vitamin D metabolism).

Peng et al. reported on a longitudinal pediatric cohort, examining sVD-level and clinical outcomes of Omicron subvariant-BA.2. Changes in immuno-clinical parameters in a sufficient sVD-level [sVD group] (*n* = 80) and insufficient sVD-levels [iVD group] (*n* = 36) were repeatedly monitored until 28 days after admission.

On day 3, higher interleukin 6, procalcitonin, and lymphocytes were observed to be significantly higher in the iVD group vs. the sVD group. On day 5, regarding the viral clearance biomarkers, the sVD group had significantly higher cycle thresholds for the N gene (32.9 vs. 26.7) and ORF1ab gene (35.2 vs. 28.3). Pneumonia lesions on computed-tomography combined with Artificial Intelligence (CT-AI) were found in 11 and six cases, respectively, in the iVD and sVD groups without significant difference at admission. Repeated CT-AI after 1–2 weeks revealed more significant improvement in lesions in the sVD group (*p* = 0.039).

This outlines how crucial it is to maintain optimal sVD-levels (cut-off 30 ng/ml), improving clinical outcomes of sub-variant BA.2 in children, thus considering the viral facet.

Hu and Xu reported on a bibliometric analysis (BA) of “dietary micronutrients related to COVID-19” (2019–2022) using the Wos-viewer on literature metadata and Citespace.

BA resulted in 170 authors in 451 journals. Most studies were published in Nutrients (61.7%) or Journal of Medical Virology (13.1%). The top three authors were Wang, Grant, and Singh. The two most co-cited references were Martineau (BMJ) and Grant (Nutrients). Mapping worldwide activities showed 417 links and 92 nodes in the cooperative network between countries/regions. The highest number of publications were the USA (186.2%), India (85.9%), and Italy (82.9%).

The keyword co-occurrence frequencies were vitamin D (302 times) and supplementation (VDD; 140 times). BA focused on vitamins D, K, and C and mechanisms (oxidative stress, ferritin, and pro-resolving mediators). They discussed the mainstream position of vitamin D in a COVID-19 context.

This study complies with Bibliometric standards (17) with explicit map visualization for each question.

As the first comprehensive examination of research on micronutrients related to COVID-19 disease using a BA-tool, outside conventional biomedical studies, it reveals vitamin D as an element of interest in COVID-19, overlooking trace elements.

To set the scene for the Research Topic by Hashemian et al., Polyunsaturated Fatty Acids (FA) play an important role as precursors to inflammatory and anti-inflammatory derivates from the ω 6 and ω 3 types. Gamma-lino-Acid (GLA) is an anti-inflammatory FA from the ω 6 family.

Authors reported on a 5-day randomized dietary trial to evaluate the effectiveness of borage oil plus syrup (BPS rich in GLA) in 60 patients with COVID-19 in an ICU. They were randomly allocated to either the BPS arm (5 ml containing 20 mg/ml GLA) or the control group with standard care (IFN-b and favipiravir). They monitored PaO_2_/FiO_2_, serum ferritin, cytokines (IL-8, IL6, and TNFα), CRP, bilirubin, ALT/AST, and PCT. Except for PaO_2_/FiO_2_, all parameters decreased significantly with BPS treatment. The suppression of serum TNF levels in the BPS group was greater than that observed in the control group. The GLA arm showed a significantly better clinical outcome (ICU length of stay).

This small-sized study inspires burgeoning GLA interest in immune-boosting in COVID-19 disease.

## Concluding remarks

Each study highlights valuable RT aspects through cohort studies, systematic reviews, and meta-analyses. They could inspire further thought on the Research Topic.

They remarkably fit within the current endemic VI, with RNA viruses targeting immune cells with inflammatory hallmarks. HIV causes long-lasting immune depression controlled by the HART spectrum. The SARS-CoV-2 variants cause successive acute clinical stages, including long COVID-19 (18).

Micronutrient status plays an important role in VI (19), especially secosteroid Vitamin D, with both genomic and non-genomic action as hot topics and eclipsing other micronutrients that are no less rich in immune multi-benefits, such as selenium, which is worth in-depth investigation (20, 21).

Vitamin D effectiveness in COVID-19 is consistently discussed and spawns challenging points (i.e., Vitamin D related to Metabolite-Ratio in lineage immune cells, genetic-polymorphisms of Vitamin D receptor, CYP2R1, CPY27B1, CYP24A1, and the interferon signaling pathway) (22–25). Answers to some aspects of these basic questions could be featured among the latest vitamin D breakthroughs during VI, in a near-future 25th workshop (vitamindworkshop.org).

## Author contributions

BR: Conceptualization, Writing—original draft, Writing—review and editing. HL: Conceptualization, Writing—review and editing. Both authors contributed to article review and editing, then approved the submitted version.
